# An Outline of New Approach to Computation with Z-Numbers Based on the Concept of Lower Prevision

**DOI:** 10.3390/e28070743

**Published:** 2026-07-01

**Authors:** Rafik Aliev, Oleg Huseynov, Aziz Nuriyev

**Affiliations:** 1 Joint MBA Program, Azerbaijan State Oil and Industry University, 20 Azadlig Ave., AZ1010 Baku, Azerbaijan; aziz.nuriyev@asoiu.edu.az; 2 Research Laboratory of Intelligent Control and Decision Making Systems in Industry and Economics, Azerbaijan State Oil and Industry University, 20 Azadlig Ave., AZ1010 Baku, Azerbaijan; oleq.huseynov@asoiu.edu.az

**Keywords:** Z-number, imprecise probability, binary operations on Z-numbers, lower and upper previsions, triangular fuzzy number

## Abstract

The concept of the Z-number was introduced to formalize partially reliable information. A Z-number represents linguistic evaluations of a random variable’s value and the associated reliability degree. The latter is defined as a fuzzy restriction on the value of a probability measure since the actual probability distribution is unknown. Lotfi Zadeh formalized an extension principle for computation with Z-numbers based on fuzzy and probabilistic arithmetic and noted that the problem of computing with Z-numbers is easy to formulate but difficult to solve. Since then, a series of theoretical studies and practical applications of Z-numbers has been proposed. However, the computational complexity of Z-numbers remains a challenge. Because the actual probability distribution is unknown, a set of probability distributions is considered, which is the main source of computational complexity. In this study, we outline a new approach to computation with Z-numbers that relies on the concept of imprecise probability. Specifically, we use a lower prevision measure (the lower envelope of a set of probability measures) as the basis for computation. The reason is a one-to-one correspondence between lower previsions and convex sets of probability measures. Experimental results show that the proposed approach reduces computational complexity compared with existing methods.

## 1. Introduction

Prof. Zadeh introduced the concept of the Z-number to formalize partially reliable information. The Z-number, denoted as Z(A, B), is an ordered tuple of fuzzy numbers *A* and *B*, encoding a linguistic (imprecise) description of a value of a random variable and the related reliability degree [1]; for example: “Air temperature will be about 30 °C; I am sure at about 80%”. The reason to use an imprecise evaluation is that the actual probability distribution of a random variable is not known. An extension principle for computation with continuous Z-numbers is introduced based on fuzzy and probabilistic arithmetic. As Zadeh noted in [1], the problem of computation with Z-numbers is significantly easier to formulate than to solve.

Since the concept’s introduction, a substantial body of work has been devoted to the theory and applications of Z-numbers [2,3,4,5,6]. From a computational point of view, the studies have developed along two main directions. One of the earliest practical schemes was proposed by Kang et al. [2], who converted a Z-number into a classical fuzzy number using fuzzy expectation. This greatly simplifies subsequent calculations, since one can then work within the standard framework of fuzzy arithmetic. However, this convenience comes at a cost: once the original two-component structure is compressed into an equivalent fuzzy representation, part of the information carried by the Z-number is inevitably lost, especially in the reliability part [2].

A different line of research follows Zadeh’s original semantics more closely and attempts to perform arithmetic through hidden probability information. In this direction, Aliev et al. proposed the arithmetic of discrete Z-numbers [3] and later extended it to the continuous case [7]. These works provided one of the first systematic frameworks for basic operations such as addition, subtraction, multiplication, and division without collapsing the Z-number into an ordinary fuzzy number at the outset. The strength of this approach is that it preserves the internal semantics of Z-information more faithfully. Its weakness is the substantially higher computational burden [3,7]. An alternative approach was proposed by Pirmuhammadi et al. [8], who developed a parametric approach to Z-numbers by representing them as pairs (A, N)—where A is triangular and N is a normal reliability function—and defining componentwise arithmetic operations. This formulation makes analytical treatment more convenient and was further used to support developments such as Z-number differentiation and Z-number initial value problems. However, it departs from hidden-distribution-based semantics and therefore follows a different interpretation of Z-number computation [8].

Further attempts to reduce the computational burden have also been proposed. Zhu et al. [9] introduced an approximate calculation method based on kernel density estimation and a utility measure of Z-numbers. However, this approach is primarily intended to reduce the number of Z-numbers to be processed in large datasets, rather than to simplify the internal structure of arithmetic operations. More recently, Li et al. [10] proposed an arithmetic framework for triangular Z-numbers with reduced calculation complexity by extending the triangular distribution used to represent hidden probabilistic information. Although this is an important step toward more efficient computation, the method remains tied to a particular distributional setting. Another important difficulty is that, in hidden-distribution-based approaches, recovering hidden probabilistic information may itself require solving optimization problems [3,7]. This is already implicit in frameworks that reconstruct admissible hidden distributions before applying arithmetic operations. This point becomes even clearer in later work. For example, Liu et al. [11] considered optimization models based on Maximum Shannon Entropy and a Genetic Algorithm for handling hidden probability distributions of discrete Z-numbers.

A review of the literature [12,13,14] shows that the main approaches to arithmetic with Z-numbers can be grouped into two directions: conversion-based methods [2] and direct arithmetic over discrete or continuous Z-numbers (e.g., [10,11]). The second direction also includes works on informativeness-preserving approaches based on specificity measures [5]. Conversion-based methods are attractive for their low computational cost, but they inevitably reduce the original Z-number information. Direct methods preserve the structure of Z-information more faithfully, albeit at the cost of considerably greater computational difficulty [15,16]. Hence, the main computational difficulty of Z-number arithmetic is clear: conversion-based approaches are simpler but lead to information loss, whereas hidden-distribution-based approaches preserve more of the original semantics but require more demanding computational procedures [2,3,11].

In a broader sense, a Z-number represents uncertainty with respect to the actual probability distribution of a random variable, which is often the case in real-world problems. Formally, this uncertainty is described by a set (mixture) of possible probability distributions, *G*, which can be constructed from *A* and *B.* In turn, studies on uncertainty about probability were initiated long ago by Boole [17], Keynes [18] (who proposed describing this uncertainty as interval estimates), and other authors. The first fundamental works in this direction were proposed by Walley [19], Kuznetsov [20], and Weichselberger [21]. Walley introduced the term “imprecise probability,” and one of the main ideas was using lower or upper previsions (which are non-additive measures). In particular, lower (or upper) previsions can be constructed as the lower (or upper) envelope of a mixture of distributions. In [15,16,22], the authors consider the relation between Z-numbers and non-additive measures.

In this paper we propose a new approach. Let us discuss the features of existing works to express the underlying motivation. In Z-numbers, uncertainty is described in forms such as “a value of probability measure $P\left(A\right)=\sum_{i=1}^{n}\mu_{A}\left(x_{i}\right)p\left(x_{i}\right)$ is B”. This implies consideration of a set of possible latent probability distributions $G=\left\{p:\sum_{i=1}^{n}\mu_{A}\left(x_{i}\right)p\left(x_{i}\right)=b∊suppB\right\}$. Thus, to conduct operation $Z_{12}=Z_{1}\circ Z_{2}$, one needs to derive a set of possible distributions $G_{12}$ related to the result $Z_{12}$ based on sets $G_{i},i=1,2$ related to the operands $Z_{i},i=1,2$. In view of this, a substantial portion of the literature—especially approaches that remain close to Zadeh’s original semantics—relies on the following computation stages:

- Construction of the set G is based on extracting latent probability distributions by solving a series of optimization problems.
- Implementing operations over a series of latent induced probability distributions $p_{i}$ in sets $G_{i},i=1,2$ to derive $G_{12}$ as a set of convolutions $p_{12}=p_{1}\circ p_{2}.$

These stages are characterized by high computational complexity.

The authors of [10] propose to reduce computational complexity by using triangular-shaped probability distributions. The idea is that one does not need to solve optimization problems because triangular distributions can be derived analytically. However, to obtain the result of operations over Z-numbers, one still needs to conduct computations over all possible pairs of distributions $p_{1}∊G_{1},p_{2}∊G_{2}$. Let us also note that as a triangular-shaped probability distribution is not usually encountered in real problems, a problem of modeling accuracy may arise.

There remains a need for a framework that preserves the two-component Z-number structure, reduces computation, and does not rely on a single parametric probability family. To address this, we propose to rely on imprecise probability theory. According to the principle of imprecise probability methods, a set of probability distributions can be described by a lower prevision (or upper prevision) without loss of generality [19]. In this work, we propose an outline of a new approach to computation with Z-numbers relying on this principle. Assume that given two Z-numbers, $Z_{1},Z_{2}$, we need to compute the result of a binary operation ∘ (e.g., a sum operation): $Z_{12}=Z_{1}\circ Z_{2}$. We propose to construct a lower prevision ${\underline{P}}_{i}$ for each set $G_{i},i=1,2$ and then to compute the resulting lower prevision ${\underline{P}}_{12}$. Then, the set of ${\underline{G}}_{12}$ can be computed based on ${\underline{P}}_{12}$. This approach reduces computational complexity as compared to the case when operation ∘ over all probability distributions in sets $G_{i},i=1,2$ is conducted. The suggested procedure is intended to reduce the internal computational burden of Z-number arithmetic without collapsing the original two-component structure of Z-information. Overall, the proposed approach is characterized by the following:

- A significant reduction in computational complexity without loss of accuracy due to one-to-one correspondence between lower prevision and a set of probability measures [19].
- A generality of computation framework stemming from the invariance of the prevision function with respect to the type of probability distribution. For computations, only probability bounds [19,23] (induced by alpha cuts of B parts) are used.

Also, the reliability $B_{12}$ of the result $Z_{12}$ may be higher than that obtained by other existing methods ($B_{12}$ decreases less given the same initial $B_{1}$ and $B_{2}$). When using the proposed method, calculations for solving problems with a large number of Z-numbers become more efficient due to a reduction in the rate of decrease in the value of B.

We provide an example comparing the proposed technique with two alternative approaches. The results show that the same accuracy is achieved with reduced computational complexity.

The potential applications of the proposed approach cover quantitative analysis problems involving operations over a series of variables under partially reliable information. In particular, this may include multi-criteria group decision-making when expert opinions on criteria importance weights and criteria values are not equally reliable. The other potential applications are engineering risk assessment, determination of reliability of sensor data, project risk analysis under incomplete information, transport system reliability, technical diagnostics, and forecasting with partially trusted expert opinions.

This paper is structured as follows: Necessary definitions are given in Section 2. In Section 3, we describe the proposed approach to operations of Z-numbers with reduced computational complexity. Numerical experiments used to illustrate the proposed approach are given in Section 4. Section 5 concludes the paper.

## 2. Materials and Methods

**Definition** **1** **([24]).** *A discrete fuzzy number. A fuzzy subset* A *of the real line* R *with membership function* $\mu_{A}:R\to \left[0,\;1\right]$ *is a discrete fuzzy number if its support is finite; i.e., there exist* $x_{1},\ldots ,x_{n}\in R$ *with* $x_{1}<x_{2}<\ldots <x_{n}$ *such that* $\mathrm{supp}\left(A\right)=\left\{x_{1},\ldots ,x_{n}\right\}$*, and there exist natural numbers* s,t *with* 1≤s≤t≤n*, satisfying the following conditions:*

*1.*  $\mu_{A}\left(x_{i}\right)=1$ *for any natural number i with* s≤i≤t*.*
*2.*  $\mu_{A}\left(x_{i}\right)\leq \mu_{A}\left(x_{j}\right)$ *for each natural number i, j with* 1≤i≤j≤s*.*
*3.*  $\mu_{A}\left(x_{i}\right)\geq \mu_{A}\left(x_{j}\right)$ *for each natural number* i,j*. with* t≤i≤j≤n.

**Definition** **2** **([25]).** *Random variables and probability distributions. A random variable,* X*, is a variable whose possible values,* x*, are numerical outcomes of a random phenomenon. Random variables are of two types: continuous and discrete. Consider a discrete random variable X with outcomes space* $X=\left\{x_{1},\ldots ,x_{n}\right\}$*. A probability of an outcome X = x_i_, denoted as* $P\left(X=x_{i}\right)$*, is defined in terms of a probability distribution. A function p is called a discrete probability distribution or a probability mass function if* $P\left(X=x_{i}\right)=p\left(x_{i}\right)$*, where* $p\left(x_{i}\right)\in \left[0,\;1\right]$ *and* $\sum_{i=1}^{n}p(x_{i})=1$*.*

**Definition** **3** **([26,27]).** *Probability measure of a discrete fuzzy number. Let A be a discrete fuzzy number and* p *be a probability distribution defined over* $X=\left\{x_{1},\ldots ,x_{n}\right\}$*. A probability measure of A, denoted as P(A), is defined as*

$$P\left(A\right)=\sum_{i=1}^{n}\mu_{A}\left(x_{i}\right)p\left(x_{i}\right).$$

Often, in real-world problems, it is not possible to determine actual probability distribution *p* due to a lack of information. Prof. Zadeh introduced the concept of the Z-number to describe imprecise information on possible probability distributions *p*.

**Definition** **4.** *A discrete* Z*-number* [1, 3]*. Given two discrete fuzzy numbers* A *and* B *such that* supp A ⊆ R *and* supp B ⊆ [0, 1], *a discrete* Z*-number is considered, denoted as* Z(A, B)*. The* Z*-number,* Z(A, B)*, represents information on a value of* X *in the following form:*

$$“\mathrm{probability}\;\mathrm{measure}\;P\left(A\right)=\sum_{i=1}^{n}\mu_{A}\left(x_{i}\right)p\left(x_{i}\right)\mathrm{is}B”.$$

As information on P(A) is imprecise, one considers a set of probability distributions of X, where an actual probability distribution for X is unknown. Denote this set of probability distributions as G:

$$G=\left\{p:\sum_{i=1}^{n}\mu_{A}\left(x_{i}\right)p\left(x_{i}\right)=b∊suppB\right\}\;,$$

where supp B is a support of B. Thus, the values b∊ supp B induce set G.

In the existing literature, various approaches are used to describe a set G of possible probability distributions *p*. If, for all $x_{i}∊X$ lower (or upper) bounds, $\underline{p}\left(x_{i}\right)$ for $p\left(x_{i}\right)$ are known, then one can define $G=\left\{p:p\left(x_{i}\right)\;\leq \underline{p}\left(x_{i}\right),{\forall x}_{i}∊X\right\}$. Denote *P* as a probability measure defined over the σ-algebra X of crisp subsets of ***X***. Denote C as a set of probability measures *P* induced by *G* (in the existing literature, such a set is referred to as a credal set [23]). The following definition applies (see, for example, [19]):

**Definition** **5** **(A lower envelope [19]).** *A lower envelope is a function* $\underline{P}:X\to \left[0,\;1\right]$*, which is defined as follows:*

$$\underline{P}\left(H\right)=\underset{P\in C}{min}P\left(H\right),\;H\subset X$$

Thus, a lower envelope is determined as a minimal probability of H⊂X. The dual-concept upper envelope is defined as $\bar{P}\left(H\right)=1-\;\underline{P}\left(H^{c}\right)$, where $H^{c}$ is the complement of H. Thus, $\underline{P}\left(H\right)\leq P\left(H\right)\leq \bar{P}\left(H\right),$ ∀H⊂X,P∈C. A lower envelope is a kind of lower probability. The latter is a special case of lower prevision. These concepts are defined for the concept of a gamble [19]. Formally, a gamble is a bounded mapping from a set of values of a random variable to a set of real numbers: f:X→R.

**Definition** **6** **(A lower prevision [19]).** *A lower prevision is a function* $\underline{P}$ *satisfying all* f,g∊F*, where* F *denotes the linear space of all gambles:*

$$\underline{P}\left(f\right)\geq \inf\left\{f\left(x\right):x∊X\right\},$$

$$\underline{P}\left(cf\right)=\underline{cP}\left(f\right),\;c∊R^{+},$$

$$\underline{P}\left(f+g\right)\geq \underline{P}\left(f\right)+\underline{P}\left(g\right).$$

A lower probability is a special case of lower prevision obtained when F is a set of all indicator functions defined over **X**. Thus, if F is a set of membership functions (when a membership function of a fuzzy set is considered as a gamble), one obtains a lower prevision as a more general measure. As lower probability can be constructed as lower envelope of probability measures of crisp sets (Definition 5), a lower prevision can be obtained in the form of a lower envelope of the probability measures of fuzzy events A:

(1) $$\underline{P}\left(A\right)=\underset{P\in C}{min}P\left(A\right),A\subset X$$

There is a one-to-one correspondence between coherent lower previsions and non-empty closed convex sets of probability measures ([19], Theorem 3.6.1). We rely on this result to propose a new approach to computations with Z-numbers using Definitions 4–6 and Formula (1).

## 3. Proposed Lower-Prevision-Based Framework

Let two Z-numbers $Z_{1}\left(A_{1},B_{1}\right),Z_{2}\left(A_{2},B_{2}\right)$ be given that describe partially reliable information on values of independent random variables $X_{1}$ and $X_{2}$. Assume we need to arrive at imprecise and partially reliable information about the value of random variable $X_{12}$ as a result of a basic arithmetic operation $\circ \in \left\{+,-,\cdot ,-\right\}$ over values of $X_{1}$ and $X_{2}$:

$$X_{12}=X_{1}\circ X_{2}.$$

We consider the problem of the computation of a result $Z_{12}\left(A_{12},B_{12}\right)$ of a basic arithmetic operation $\circ \in \left\{+,-,\cdot ,-\right\}$ over two Z-numbers $Z_{1}\left(A_{1},B_{1}\right),Z_{2}\left(A_{2},B_{2}\right)$:

$$Z_{12}=Z_{1}\circ Z_{2}.$$

The process of solving the problem in question consists of the following:

(1) Computation of the A part of the result:$\;A_{12}$ is a fuzzy number computed on the basis of fuzzy numbers $A_{1},A_{2}$ using fuzzy arithmetic $A_{12}=A_{1}\circ A_{2}$ [24].
(2) Construction of sets of $G_{l},l=1,2$ by extracting of probability distributions for Z-numbers $Z_{l}=\left(A_{l},B_{l}\right),l=1,2$: This implies that for each $b\in supp{\;B}_{l}$ we need to find probability distribution $p_{i}$ satisfying:
  $$b=\sum_{i=1}^{n}\mu_{A_{l}}\left(x_{i}\right)p\left(x_{i}\right).$$
  Thus, a set of values $b_{i}$ induces a set of probability distributions p.
(3) Construction of set $G_{12}$ on the basis of sets $G_{l},l=1,2$: A set $G_{12}$ is a set of convolutions, $p_{12}$, induced by sets $G_{l},l=1,2$: $G_{12}=\left\{r=p\circ q:p\in G_{1},q\in G_{2}\right\}$. It is well-known that convolution r=p∘q is determined as follows [25]:
  $$r\left(x_{12}\right)=\sum_{x=x_{1}*x_{2}}p(x_{1})q(x_{2}),$$
  In this study, independence is assumed as a baseline case. Dependence-aware extensions may be considered in future research.
(4) Computation of the B part of the result: $B_{12}$ is a fuzzy number computed on the basis of fuzzy number $A_{12}$ and probability distributions $r\in G_{12}$. This implies that each $r\in G_{12}$ induces $b_{12}$:
  $$b_{12}=\sum_{i=1}^{n}\mu_{A_{12}}\left(x_{12i}\right)\;r\left(x_{12i}\right).$$

In order to reduce the computational complexity of operations with Z-numbers, we propose not dealing directly with sets $G_{l},l=1,2$. Instead, we propose to use the lower previsions as lower envelopes of the corresponding sets $C_{l},l=1,2$ (recall that C is a set of probability measures P induced by set G of probability distributions).

Below, we describe the main stages of the proposed approach.

**Stage 1. Computation of A part** $A_{12}$**.** At this stage, a fuzzy number $A_{12}$ is computed based on fuzzy numbers $A_{l},l=1,2$.

To make arithmetic operations computable, we replace each first component by a finite discrete approximation:

$$A_{1}^{d}=\{(x_{i}^{\left(1\right)},\mu_{i}^{\left(1\right)}){\}}_{i=1}^{n_{1}},{\;A}_{2}^{d}=\{(x_{j}^{\left(2\right)},\mu_{j}^{\left(2\right)}){\}}_{j=1}^{n_{2}}.$$

Here, $x_{i}^{\left(1\right)}=x_{1i}$ and $x_{j}^{\left(2\right)}=x_{2j}$ are discretization nodes, while $\mu_{i}^{\left(1\right)}$ and $\mu_{j}^{\left(2\right)}$ are the corresponding membership values inherited from the original fuzzy numbers. Then, $A_{12}=A_{1}\circ A_{2}$ can be computed.

**Stage 2. Extraction of probability distributions for Z-numbers** $Z_{l}=\left(A_{l},B_{l}\right),l=1,2$ **and construction of lower previsions.**

For Z-numbers $Z_{l}=\left(A_{l},B_{l}\right),l=1,2$, the corresponding sets $G_{1}=\left\{p\right\},\;G_{2}=\left\{q\right\}$ of probability distributions are constructed on the basis of $\left(A_{l},B_{l}\right),l=1,2$. Thus, latent probability distributions are induced:

$$p=\left(p_{1},\ldots ,p_{n_{1}}\right),q=\left(q_{1},\ldots ,q_{n_{2}}\right),$$

subject to

$$p_{i}\geq 0,\sum_{i=1}^{n_{1}}p_{i}=1,$$

$$q_{j}\geq 0,\sum_{j=1}^{n_{2}}q_{j}=1.$$

Here, $p_{i}=p\left(x_{i}^{\left(1\right)}\right)$ and $q_{j}=q\left(x_{j}^{\left(2\right)}\right)$.

These distributions represent possible latent probability patterns on the discretized supports of $A_{1}$ and $A_{2}$. To keep the probabilistic layer aligned with the first components, the compatibility conditions are imposed [1]:

$$\sum_{i=1}^{n_{1}}x_{i}^{\left(1\right)}p_{i}=C_{1},\sum_{j=1}^{n_{2}}x_{j}^{\left(2\right)}q_{j}=C_{2}.$$

The constants $C_{1}$ and $C_{2}$ may be chosen either as the centroids of the original triangular fuzzy numbers

$$C_{1}=\frac{l_{1}+m_{1}+r_{1}}{3},C_{2}=\frac{l_{2}+m_{2}+r_{2}}{3},$$

or as the centroids of their discrete approximations:

$$C_{1}=\frac{\sum_{i=1}^{n_{1}}x_{i}^{\left(1\right)}\mu_{i}^{\left(1\right)}}{\sum_{i=1}^{n_{1}}\mu_{i}^{\left(1\right)}},C_{2}=\frac{\sum_{j=1}^{n_{2}}x_{j}^{\left(2\right)}\mu_{j}^{\left(2\right)}}{\sum_{j=1}^{n_{2}}\mu_{j}^{\left(2\right)}}.$$

For each latent distribution, we define the induced probability measures of fuzzy events:

$$P_{p}\left(A_{1}\right)=\sum_{i=1}^{n_{1}}\mu_{i}^{\left(1\right)}p_{i},P_{q}\left(A_{2}\right)=\sum_{j=1}^{n_{2}}\mu_{j}^{\left(2\right)}q_{j}.$$

These quantities measure how strongly the latent distributions are concentrated in the high-valued membership regions of $A_{1}$ and $A_{2}$.

Now recall that the values of $P_{p}\left(A_{1}\right)$ and ${\;P}_{q}\left(A_{2}\right)$ are restricted by α-cuts of the second components of $Z_{i}=\left(A_{i},B_{i}\right),i=1,2$:

$$[B_{1}{]}^{\alpha }=[L_{1}(\alpha ),U_{1}(\alpha )],[B_{2}{]}^{\alpha }=[L_{2}(\alpha ),U_{2}(\alpha )].$$

This implies that at level α, these intervals define the following sets:

$$K_{1}^{\alpha }=\left\{p:\;\;p_{i}\geq 0,\;\;\sum_{i=1}^{n_{1}}p_{i}=1,\;\;\sum_{i=1}^{n_{1}}x_{i}^{\left(1\right)}p_{i}=C_{1},\;\;L_{1}\left(\alpha \right)\leq P_{p}\left(A_{1}\right)=\sum_{i=1}^{n_{1}}\mu_{i}^{\left(1\right)}p_{i}\leq U_{1}\left(\alpha \right)\right\},$$

$$K_{2}^{\alpha }=\left\{q:\;\;q_{j}\geq 0,\;\;\sum_{j=1}^{n_{2}}q_{j}=1,\;\;\sum_{j=1}^{n_{2}}x_{j}^{\left(2\right)}q_{j}=C_{2},\;\;L_{2}\left(\alpha \right)\leq P_{q}\left(A_{2}\right)=\sum_{j=1}^{n_{2}}\mu_{j}^{\left(2\right)}q_{j}\leq U_{2}\left(\alpha \right)\right\}.$$

Note that $K_{i}^{\alpha }$ is an α-cut of $G_{i}$, i=1,2. For each set K, a lower prevision $\underline{P}\left(A\right)$ is computed based on Formula (1). For example, one has for $K_{1}^{\alpha }$:

$$\underline{P}\left(A_{1}\right)={min}_{p\epsilon K_{1}^{\alpha }}\left(P_{p}\left(A_{1}\right)\right).$$

**Stage 3. Construction of lower prevision of resulting Z-number based on lower previsions for Z-numbers** $Z_{i}=\left(A_{i},B_{i}\right),i=1,2.$

Given the lower previsions $\underline{P}\left(A_{i}\right)$ obtained for $Z_{i}=\left(A_{i},B_{i}\right),i=1,2$, the lower prevision of $A_{12}$ (A part of Z-number $Z_{12}$), $\underline{P}\left(A_{12}\right)$, is computed. For each pair of nodes $\left(x_{i}^{\left(1\right)},x_{j}^{\left(2\right)}\right)$, we compute the pairwise outcome

$$z_{ij}=x_{i}^{\left(1\right)}\circ x_{j}^{\left(2\right)}.$$

All distinct values $z_{ij}$ can be considered as set ${\left\{z_{s}\right\}}_{s=1}^{N}$, i.e., the set of the discrete support of the first component of the result. These points $z_{s}$ are all possible outcomes obtained by applying the arithmetic operation to pairs of discretization nodes.

For any admissible pair,

$$p\in K_{1}^{\alpha },q\in K_{2}^{\alpha },$$

the latent distribution of the result is defined by

$$r\left(z_{s}\right)=\sum_{z_{ij}=z_{s}}p_{i}q_{j}.$$

So $r\left(z_{s}\right)$ is the probability assigned to the result point $z_{s}$ by the latent distributions p and q. $p_{i}q_{j}$ corresponds to the standard independent combination of the two latent distributions.

Let $A_{12}=A_{1}\circ A_{2}$ be the first component of the result and $A_{12}^{d}=\{(z_{s},\mu_{s}^{\left(12\right)}){\}}_{s=1}^{N}$ be its discretized representation. Then, the inherited reliability of the result is defined by

$$P_{p,q}^{\left({}^{\circ}\right)}\left(A_{12}\right)=\sum_{s=1}^{N}\mu_{s}^{\left(12\right)}r\left(z_{s}\right).$$

This formula has a simple meaning: each point $z_{s}$ of the resulting support has two attributes—its latent probability $r\left(z_{s}\right)$ and its membership value $\mu_{s}^{\left(12\right)}$. The inherited reliability is just the weighted average of these membership values with respect to the latent distribution of the result.

The same quantity can be written directly in terms of the original node pairs. Indeed,

$$P_{p,q}^{\left({}^{\circ}\right)}\left(A_{12}\right)=\overset{n_{1}}{\underset{i=1}{\sum }}\overset{n_{2}}{\underset{j=1}{\sum }}\mu_{A_{12}}\left(x_{i}^{\left(1\right)}\circ x_{j}^{\left(2\right)}\right)p_{i}q_{j}.$$

This leads naturally to the weight matrix:

$$M^{\left({}^{\circ}\right)}=\left(\mu_{ij}^{\left({}^{\circ}\right)}\right)\in R^{n_{1}\times n_{2}},\mu_{ij}^{\left({}^{\circ}\right)}=\mu_{A_{12}}\left(x_{i}^{\left(1\right)}\circ x_{j}^{\left(2\right)}\right).$$

The entries of $M^{\left({}^{\circ}\right)}$ can be called weights because they tell us how strongly each pairwise outcome agrees with the first component of the result. Each of them is simply the membership value of the pairwise outcome $x_{i}^{\left(1\right)}\circ x_{j}^{\left(2\right)}$ in the fuzzy number $A_{12}$, with the following notation:

$$P_{p,q}^{\left({}^{\circ}\right)}\left(A_{12}\right)=p^{T}M^{\left({}^{\circ}\right)}q.$$

So the whole mechanism is

$$\left(p,q\right)\;\;⟶\;\;r\left(z_{s}\right)\;\;⟶\;\;P_{p,q}^{\left({}^{\circ}\right)}\left(A_{12}\right),$$

where $r\left(z_{s}\right)$ is the latent distribution of the result; $M^{\left({}^{\circ}\right)}$ is the matrix that contains the membership grades of all pairwise outcomes; and $p=(p_{1},\ldots ,p_{n_{1}}{)}^{T}$ and $q=(q_{1},\ldots ,q_{n_{2}}{)}^{T}$ denote hidden probability distributions on the discretized supports of $A_{1}$ and $A_{2}$, respectively.

Finally, the second component of the resulting Z-number is reconstructed level by level:

$$[B_{12}^{\left({}^{\circ}\right)}{]}^{\alpha }=\left[{\underset{¯}{b}}_{12}^{\left({}^{\circ}\right)}(\alpha ),\;\;{\bar{b}}_{12}^{\left({}^{\circ}\right)}(\alpha )\right],$$

where

(2) $${\underset{¯}{b}}_{12}^{\left({}^{\circ}\right)}\left(\alpha \right)=\underset{p\in K_{1}^{\alpha },\;\;q\in K_{2}^{\alpha }}{\min}p^{T}M^{\left({}^{\circ}\right)}q$$

(3) $${\bar{b}}_{12}^{\left({}^{\circ}\right)}\left(\alpha \right)=\underset{p\in K_{1}^{\alpha },\;\;q\in K_{2}^{\alpha }}{max}p^{T}M^{\left({}^{\circ}\right)}q$$

It can easily be shown that distributions p and q obtained as solutions of problem (2) underly lower previsions:

$$\underline{P}\left(A_{1}\right)={min}_{p\epsilon K_{1}^{\alpha }}\left(P_{p}\left(A_{1}\right)\right)=L_{1}\left(\alpha \right),$$

$$\underline{P}\left(A_{2}\right)={min}_{q\epsilon K_{2}^{\alpha }}\left(P_{q}\left(A_{2}\right)\right)=L_{2}\left(\alpha \right).$$

In turn, ${\underset{¯}{b}}_{12}^{\left({}^{\circ}\right)}\left(\alpha \right)$ is a lower prevision $\underline{P_{p,q}^{\left({}^{\circ}\right)}}\left(A_{12}\right)$. Thus, lower previsions $\underline{P}\left(A_{i}\right)$ obtained for $Z_{i}=\left(A_{i},B_{i}\right),i=1,2$ induce the lower prevision of $A_{12}$ (A part of Z-number $Z_{12}$), $\underline{P}\left(A_{12}\right)$. Analogously, upper probabilities $\bar{P}\left(A_{i}\right)$ induce upper probability $\bar{P}\left(A_{12}\right)$. Based on $\underline{P}\left(A_{12}\right)$ and $\bar{P}\left(A_{12}\right)$, the corresponding credal set $K_{12}^{\alpha }$—and, as a result, the $G_{12}$ set—are constructed.

**Stage 4. Computation of B part of Z-number** $Z_{12}$ **.**

$B_{12}$ (B part of Z-number $Z_{12}$) is computed by using $A_{12}$ and $G_{12}$ according to Definition 4. Thus, Z-number $Z_{12}^{\left({}^{\circ}\right)}=\left(A_{12},B_{12}^{\left({}^{\circ}\right)}\right)$ is computed, where the first component comes from the arithmetic operation applied to $A_{1}$ and $A_{2}$, while the second one is reconstructed from all inherited reliability values generated by admissible latent distributions.

**Figure 1:**
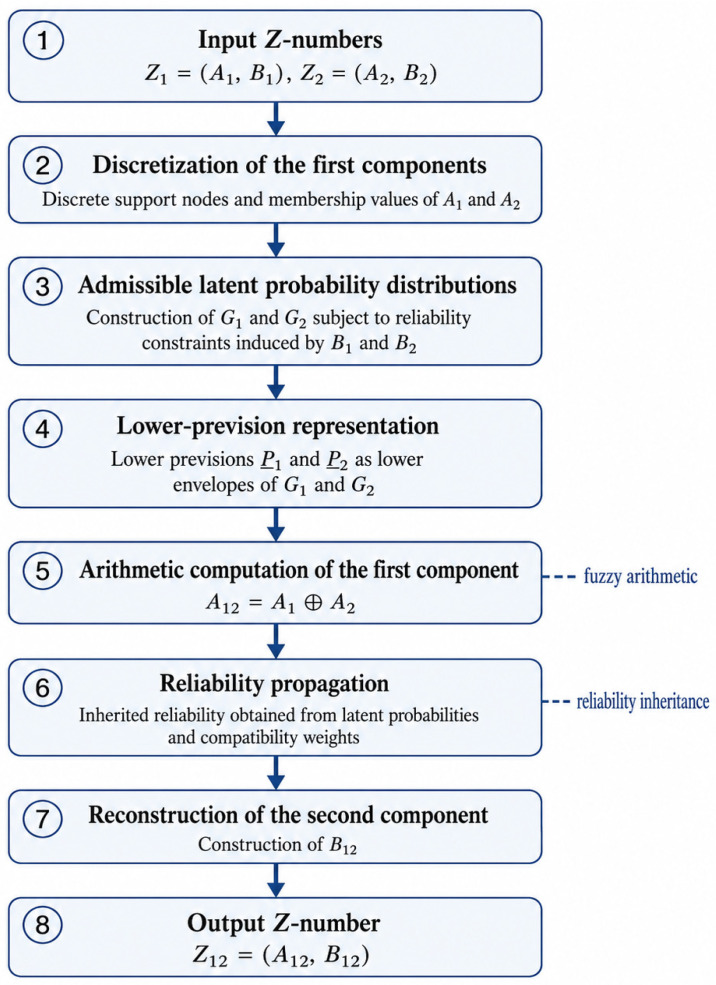
The flowchart of the computation framework.

The flowchart of the computation framework is given in Figure 1.

**Figure 2:**
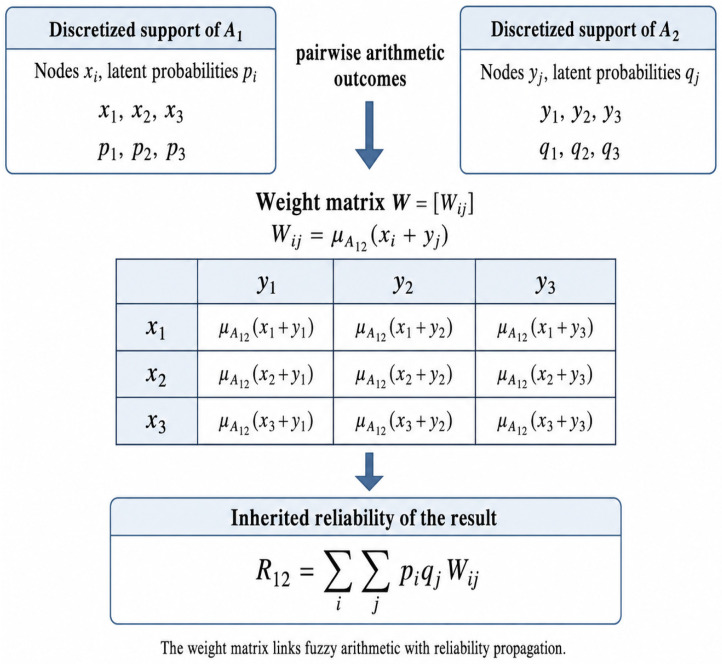
Mechanism for computing the weight matrix.

For a more detailed illustrative description, the mechanism for computing the weight matrix constructed at Stage 3 is shown in Figure 2.

Let us discuss the features of the works proposed in [3,10] as compared with the newly proposed approach. In [3], a binary operation $Z_{12}{(A}_{12},B_{12})=Z_{1}\circ Z_{2}$ is implemented as follows:

(1) $A_{12}$ is computed based on fuzzy arithmetic: $A_{12}=A_{1}+A_{2}$.
(2) For $Z_{l},$ the sets $G_{l}=\left\{p_{l}:\sum_{i=1}^{n}\mu_{A_{l}}\left(x_{i}\right)p_{l}\left(x_{i}\right)=b_{l}∊suppB_{l}\right\},\;l=1,2$ are determined. Probability distributions $p_{l}$ are found by solving linear optimization problems:
  $$\mathrm{Find}\;p_{l}\mathrm{such}\;\mathrm{that}\;\sum_{i=1}^{n}\mu_{A_{l}}\left(x_{i}\right)p_{l}\left(x_{i}\right)=b_{l}\;s.t.\;\sum_{i=1}^{n}p_{l}\left(x_{i}\right)=1,\;p_{l}\left(x_{i}\right)\geq 0.$$
  Thus, one needs to solve $n_{l}\;$ optimization problems ($n_{l}$ is the number of basic values $b_{l})$.
(3) Given $G_{l},\;l=1,2$, one needs to construct $G_{12}$ as a set of convolutions:
  $$G_{12}=\left\{p_{12}:p_{12}\left(x\right)=\sum_{x=x_{1}+x_{2}}p_{1}\left(x_{1}\right)p_{2}\left(x_{2}\right),{p_{l}∊G}_{l},\;l=1,2\right\}.$$
  Thus, one will have to compute $n_{1}n_{2}$ convolutions $p_{12}$.
(4) $B_{12}$ is computed based on $A_{12}$ and $G_{12}$:
  $$suppB_{12}=\left\{b_{12}:{b_{12}=P(A}_{12})=\sum_{i=1}^{n}\mu_{A_{12}}\left(x_{i}\right)p_{12}\left(x_{i}\right),p_{12}{∊G}_{12}\right\}.$$

The main sources of computational complexity are stages 2 and 3. Stage 3 implies construction of array of $n_{1}n_{2}$ convolutions $p_{12}$. For example, if one considers at least $n_{1}=n_{2}=3$ distributions in each $G_{l}$, l=1,2, then 9 convolutions $p_{12}$ are constructed. In contrast, we propose solving the following problems (which are not computationally complex):

- To compute one lower prevision $\underline{P}\left(A_{l}\right)$ for each set $G_{l}$.
- To derive a lower prevision $\underline{P}\left(A_{12}\right)$ based on lower previsions $\underline{P}\left(A_{1}\right)$, $\underline{P}\left(A_{2}\right)$.
- To construct $G_{12}$ based on $\underline{P}\left(A_{12}\right)$.

Indeed, we consider *one combination of two lower previsions* $\underline{P}\left(A_{l}\right)$ *only vs. nine or more combinations of three or more distributions* ${p_{l}∊G}_{l},\;l=1,2$. We do this only when deriving the calculation formula for part *B* of the result. After that, we work with the formula that already accounts for the distribution. The previous approach required working with distributions at each operation.

Moreover, relying on the proposed idea, in the next section, we show that if the components of Z-numbers $Z_{1},Z_{2}$ are triangular fuzzy numbers, then the $B_{12}$ part of $Z_{12}=Z_{1}+Z_{2}$ can be described directly viaan analytical formula as a triangular fuzzy number without solving computationally complex problems in stages 2–3.

The work proposed in [10] allows for a significant reduction in computational complexity. This is attained due to consideration of one specific type of distribution, namely, triangular-shaped probability distributions. The basic value b∊suppB is determined as follows:

$$\int_{-\infty }^{\infty }\mu_{A}\left(x\right)p\left(x\right)dx=b$$

As membership function $\mu_{A}$ and latent distribution p are triangular-shaped, the latter can be derived analytically (without solving optimization problem). Further, the basic value of the result $b_{12}∊suppB_{12}$ is determined as follows:

$$b_{12}=\int_{-\infty }^{\infty }\mu_{A_{12}}\left(x\right)p_{12}\left(x\right)dx$$

where $p_{12}$ is convolution. As latent distributions $p_{1}$ and $p_{2}$ of operands $Z_{1}$ and $Z_{2}$ are triangular-shaped, the integral above can be described analytically (without need for numerical integration). However, one still needs to deal with a set of latent distributions $p_{1}$ and $p_{2}$ directly to consider all possible pairs $p_{1}∊G_{1},p_{2}∊G_{2}$. Also note that a triangular-shaped probability distribution is not usually found in real problems. In view of this, approximation issues may arise.

## 4. Numerical Experiments and Comparative Analysis

Let us consider numerical experiments to evaluate the proposed approach. For example, we can explore cases involving Z-numbers with triangular fuzzy number-based A and B parts. The choice of triangular fuzzy numbers to express parts A and B is due to their properties—which combine a balance between computational efficiency and the ability to represent uncertainty—being intuitive and easier to interpret, aiding decision-makers in understanding the results, and representing diverse types of uncertainty criteria across different spheres [28,29,30,31,32].

### 4.1. Addition of Two Z-Numbers

We consider two Z-numbers $Z_{1}=\left(A_{1},B_{1}\right),Z_{2}=\left(A_{2},B_{2}\right),$ where the first components are triangular fuzzy numbers and can be presented in the form below:

$$A_{1}=\left(m_{1}-h_{1},\;\;m_{1},\;\;m_{1}+h_{1}\right),A_{2}=\left(m_{2}-h_{2},\;\;m_{2},\;\;m_{2}+h_{2}\right),$$

and the second components are triangular fuzzy numbers:

$$B_{1}=\left(u_{1},v_{1},w_{1}\right),B_{2}=\left(u_{2},v_{2},w_{2}\right).$$

We apply 3-point discretization of A with only the three vertices, and 3-α levels for B $\alpha \in \left\{0,\;\;0.5,\;\;1\right\}.$ The 3-point scheme is used as the minimal analytically tractable discretization that preserves the left endpoint, modal value, and right endpoint of a triangular fuzzy number. Higher-order discretization can provide more accurate approximations but lead to more complex expressions. 

1. Discretization of the first components

For each $A_{i}$, we take the three points

$$x_{1}^{\left(i\right)}=m_{i}-h_{i},x_{2}^{\left(i\right)}=m_{i},x_{3}^{\left(i\right)}=m_{i}+h_{i},$$

with membership values being (0,1,0).

So, the discrete approximation is

$$A_{i}^{d}=\left\{\left(m_{i}-h_{i},0\right),\;\;\left(m_{i},1\right),\;\;\left(m_{i}+h_{i},0\right)\right\}.$$

2. Latent probability distributions

Because the first components are symmetric, we use symmetric latent distributions:

$$p=\left(a,\;\;1-2a,\;\;a\right),q=\left(d,\;\;1-2d,\;\;d\right),$$

with

$$0\leq a\leq \frac{1}{2},0\leq d\leq \frac{1}{2}.$$

Here:

- *a* is the probability mass assigned to each extreme point of $A_{1}$;
- 1 − 2*a* is the mass assigned to the center;
- *d* and 1 − 2*d* play the same role for $A_{2}$.

3. Induced reliability measures

Since the membership values are (0,1,0), we get

$$m_{A_{1}}\left(p\right)=0\cdot a+1\cdot \left(1-2a\right)+0\cdot a=1-2a,$$

$$m_{A_{2}}\left(q\right)=0\cdot d+1\cdot \left(1-2d\right)+0\cdot d=1-2d.$$

So, the induced reliabilities are simply

$$m_{A_{1}}\left(p\right)=1-2a,m_{A_{2}}\left(q\right)=1-2d.$$

4. The first component of the sum

The sum of the first two components is again a symmetric triangular fuzzy number:

$$A_{12}=A_{1}+A_{2}=\left(m_{1}+m_{2}-\left(h_{1}+h_{2}\right),\;\;m_{1}+m_{2},\;\;m_{1}+m_{2}+\left(h_{1}+h_{2}\right)\right).$$

5. Weight matrix for the 3-point scheme

For the standard simple case $h_{1}=h_{2}$, the weight matrix is

$$M=\left(\begin{array}{ccc} 0 & \frac{1}{2} & 1\\ \frac{1}{2} & 1 & \frac{1}{2}\\ 1 & \frac{1}{2} & 0 \end{array}\right).$$

Then, the reliability of the sum A_12_ (inherited) is

$$m_{12}\left(p,q\right)=p^{T}Mq.$$

Substituting $p=\left(a,1-2a,a\right),q=\left(d,1-2d,d\right),$ we obtain the following expression:

$$m_{12}\left(p,q\right)=1-a-d+2ad$$

This is the key analytical formula for the 3-point scheme.

6. Three levels of B

For a triangular fuzzy number

$$B_{i}=\left(u_{i},v_{i},w_{i}\right),$$

the three chosen α-cuts are as follows:

- At α=0: $\left[B_{i}{]}^{0}=[u_{i},w_{i}\right]$.
- At α=0.5: ${\left[B_{i}\right]}^{0.5}=\left[\frac{u_{i}+v_{i}}{2},\;\;\frac{v_{i}+w_{i}}{2}\right]$.
- At α=1: $[B_{i}{]}^{1}=[v_{i},v_{i}].$

Let $[B_{1}{]}^{\alpha }=[L_{1}(\alpha ),U_{1}(\alpha )],[B_{2}{]}^{\alpha }=[L_{2}(\alpha ),U_{2}(\alpha )].$

Because

$$m_{A_{1}}\left(p\right)=1-2a,m_{A_{2}}\left(q\right)=1-2d,$$

the constraints at the α-level become

$$L_{1}\left(\alpha \right)\leq 1-2a\leq U_{1}\left(\alpha \right),L_{2}\left(\alpha \right)\leq 1-2d\leq U_{2}\left(\alpha \right).$$

Hence,

$$\frac{1-U_{1}(\alpha )}{2}\leq a\leq \frac{1-L_{1}(\alpha )}{2},\frac{1-U_{2}(\alpha )}{2}\leq d\leq \frac{1-L_{2}(\alpha )}{2}.$$

7. Analytical formula for each level of $B_{12}$

Since $\;m_{12}\left(p,q\right)=1-a-d+2ad$ is decreasing in both *a* and *d* on the admissible range, we get:

- The *minimum* at the upper bounds of a and d;
- The *maximum* at the lower bounds of a and d.

After substitution, we obtain

$${\left[B_{12}\right]}^{\alpha }=\left[\frac{1+L_{1}(\alpha )L_{2}(\alpha )}{2},\;\;\frac{1+U_{1}(\alpha )U_{2}(\alpha )}{2}\right],\alpha \in \left\{0,0.5,1\right\}.$$

8. Formulas for the three levels

In α = 0, the *L*_1_ (0) = *u*_1_, *U*_1_ (0) = *w*_1_, *L*_2_ (0) = *u*_2_, *U*_2_ (0) = *w*_2_; hence, we obtain $B_{12}^{0}=\;\left[\frac{1+u_{1}u_{2}}{2},\;\;\frac{1+w_{1}w_{2}}{2}\right]$.

In α = 0.5, the $L_{1}\left(0.5\right)=\frac{u_{1}+v_{1}}{2}$, $U_{1}\left(0.5\right)=\frac{v_{1}+w_{1}}{2}$, $L_{2}\left(0.5\right)=\frac{u_{2}+v_{2}}{2}$, $U_{2}\left(0.5\right)=\frac{v_{2}+w_{2}}{2}$; hence, we obtain $B_{12}^{0.5}=\left[\frac{1+\left(\frac{u_{1}+v_{1}}{2}\right)\left(\frac{u_{2}+v_{2}}{2}\right)}{2},\;\;\frac{1+\left(\frac{v_{1}+w_{1}}{2}\right)\left(\frac{v_{2}+w_{2}}{2}\right)}{2}\right]$ or $B_{12}^{0.5}=\left[\frac{1}{2}+\frac{\left(u_{1}+v_{1})(u_{2}+v_{2}\right)}{8},\;\;\frac{1}{2}+\frac{\left(v_{1}+w_{1})(v_{2}+w_{2}\right)}{8}\right]$.

In α = 1, the *L*_1_(1) = *U*_1_(1) = *v*_1_, *L*_2_ (1) = *U*_2_ (1) = *v*_2_, and

$$B_{12}^{1}=\left[\frac{1+v_{1}v_{2}}{2},\;\;\frac{1+v_{1}v_{2}}{2}\right]$$

The result is a single point.

9. Final formula for the sum of two Z-numbers

For *Z*_1_ + *Z*_2_ = *Z*_12_(*A*_12_, *B*_12_), where

A_12_ = (m_1_ + m_2_ − (h_1_ + h_2_), m_1_ + m_2_, m_1_ + m_2_ + (h_1_ + h_2_)),

the second component $B_{12}$ is defined by the three cuts

$${\left[B_{12}{]}^{0},[B_{12}{]}^{0.5},[B_{12}\right]}^{1}$$

given above.

### 4.2. Numerical Example

Let *Z*_1_ = ((1,2,3), (0.7,0.8,0.9)), and *Z*_2_ = ((7,8,9), (0.4,0.5,0.6)).

Here, we have for *A*_1_ = (1,2,3), *m*_1_ =2, *h*_1_ = 1, and for *A*_2_ = (7,8,9), *m*_2_ =8, *h*_2_ = 1.

So A_12_ = (1 + 7,2 + 8,3 + 9) = (8,10,12).

For B, we have the following:

For α = 0—$B_{1}^{0}=\left[0.7,\;0.9\right],B_{2}^{0}=\left[0.4,\;0.6\right].$

So, $B_{12}^{0}=\left[\frac{1+0.7\cdot 0.4}{2},\;\;\frac{1+0.9\cdot 0.6}{2}\right]$, and after computation,—0.7 × 0.4=0.28, 0.9 × 0.6 = 0.54.

$$B_{12}^{0}=\left[\frac{1.28}{2},\;\;\frac{1.54}{2}\right]=\left[0.64,\;\;0.77\right].$$

For α = 0.5,

$$B_{1}^{0.5}=\left[\frac{0.7+0.8}{2},\frac{0.8+0.9}{2}\right]=\left[0.75,0.85\right],$$

$$B_{2}^{0.5}=\left[\frac{0.4+0.5}{2},\frac{0.5+0.6}{2}\right]=\left[0.45,0.55\right].$$

So, $B_{12}^{0.5}=\left[\frac{1+0.75\cdot 0.45}{2},\;\;\frac{1+0.85\cdot 0.55}{2}\right]$, and after computation,

$$B_{12}^{0.5}=\left[\frac{1.3375}{2},\;\;\frac{1.4675}{2}\right]=\left[0.66875,\;\;0.73375\right]$$

For α = 1, $B_{1}^{1}=\left[0.8,0.8\right],B_{2}^{1}=\left[0.5,0.5\right].$

So, $B_{12}^{1}=\left[\frac{1+0.8\cdot 0.5}{2},\;\;\frac{1+0.8\cdot 0.5}{2}\right]$, and we obtain $B_{12}^{1}=\left[0.7,0.7\right]$.

So, the sum is Z_1_ + Z_2_ = ((8,10,12), B_12_), and B_12_ is described by $B_{12}^{0}=\left[0.64,\;\;0.77\right]$,

$B_{12}^{0.5}=\left[0.66875,\;\;0.73375\right]$, and $B_{12}^{1}=\left[0.7,0.7\right]$.

And in the form of a triangular fuzzy number approximation, B_12_ = (0.64, 0.7, 0.77), resulting in Z_12_ = (8,10,12) (0.64, 0.7, 0.77).

### 4.3. Comparison of Results and Complexity Analysis

Let us conduct a comparison of the proposed approach with the approaches developed in works [3,10]. In both works, the computation of a sum $Z_{12}=Z_{1}+Z_{2}$ of two triangular Z-numbers, *Z*_1_ = (*A*_1_, *B*_1_) and *Z*_2_ = (*A*_2_, *B*_2_), is considered, where the components are TFNs:

*Z*_1_ = ((1, 2, 3), (0.7, 0.8, 0.9)), *Z*_2_ = ((7, 8, 9), (0.4, 0.5, 0.6)).

In [3], normal pdfs are used in this example. The result is $Z_{12\left[3\right]}=\left(\left(8,10,12\right),\left(0.62,\;0.71,\;0.79\right)\right)$. In [10], triangular distributions are used to facilitate computations. The obtained result is $Z_{12[10]}=\left(\left(8,10,12\right),\left(0.674,\;0.692,\;0.712\right)\right)$. The result obtained by the proposed approach is $Z_{12}=\left(\left(8,10,12\right),\left(0.64,\;0.7,\;0.77\right)\right)$. As one can see, the *A* parts coincide, and the *B* parts differ slightly.

Mean absolute percentage error (MAPE) between the *B* part of the result of the proposed approach $Z_{12}$ and that of the approach in [3] is $MAPE\left(B_{12\left[3\right]},B_{12}\right)=2.6\%$. The MAPE for the results obtained by the approach proposed in [10] is $MAPE\left(B_{12\left[8\right]},B_{12}\right)=4.7\%$. Thus, the proposed approach provides the same accuracy as the approaches proposed in [3,10]. At the same time, in the proposed approach, it is not required to assume a type of probability distribution.

For Python 3.12 code realizing approaches from [3,10], the execution time is 0.0560225050 and 0.049157 s, respectively. For the suggested approach, the execution time is 0.0000384460.

Let us also conduct comparisons for computation with four Z-numbers. Consider the addition of the following Z-numbers:

*Z*_1_ = ((1.0, 2.0, 3.0), (0.7, 0.8, 0.9)),

*Z*_2_ = ((7.0, 8.0, 9.0), (0.4, 0.5, 0.6)),

*Z*_3_ = ((2.0, 3.0, 4.0), (0.6, 0.7, 0.8)),

*Z*_4_ = ((4.0, 5.0, 6.0), (0.5, 0.6, 0.7)).

The proposed approach provides the result as *Z_proposed_* = ((14, 18, 22), (0.67166667, 0.7245, 0.78685)). The results are based on approaches proposed in [3,10]: *Z*_[10]_ = ((14, 18, 22), (0.65051494, 0.65385742, 0.65732196)); *Z*_[3]_ = ((14.0, 18.0, 22.0), (0.57, 0.61, 0.67)). Thus, the proposed approach provides higher reliability value.

The computational complexity of the approach proposed in [10] is O(Q + r), where Q denotes the cost of the numerical procedure used to construct the resulting reliability component, and r is the number of alpha levels. For a fixed triangular input and fixed numerical settings, this becomes O(1).

The computational complexity of the approach proposed in [3] is O(CLP(n) + m2n2), where n is the number of discretization points of A, m is the number of discretization points of B, and CLP(n) is the cost of solving one LP problem.

The computational complexity of the proposed analytical 3-point/3-alpha method is O(r). Since r = 3 in the considered implementation, the complexity is O(1).

The comparison results are given in Table 1.

The method proposed in [10] has a virtually constant computational structure; its actual execution time is determined by the numerical procedures used to construct the B component of the resulting Z-number. In the case of the approach indicated in [3], the number of convolution operations with 3-point discretization is small; however, the reconstruction of latent probability distributions requires solving LP problems, and it is the costs associated with the LP solver that dominate when the input data size is small. Therefore, the computation times for the methods [3,10] in this small example are similar. In contrast, the proposed analytical 3-point/3-alpha scheme uses neither numerical integration nor LP reconstruction of probability distributions but calculates the B12 component directly using formulas. This explains the significantly shorter execution time.

## 5. Discussion and Conclusions

In a nutshell, a Z-number represents uncertainty with respect to the actual probability distribution of a random variable, which is often the case in real-world problems. Formally, this uncertainty is described by a set (mixture) of possible probability distributions. According to the imprecise probability concept of Walley, the set of probability distributions may be captured by a lower prevision measure. In this work, we propose an outline of a new approach to computation with Z-numbers relying on this principle. This allows for the reduction in the complexity of operations over Z-numbers without imposing restrictions on a type of probability distribution. We provide an example for the computation of a sum of two Z-numbers. The results of the comparison of the proposed approach with two alternative approaches prove the validity of the former.

In the proposed approach, the induced reliability measure is directly related to the central probability mass of the latent distribution. A weight matrix plays a key role in this mechanism. Each element of the matrix reflects the degree of membership of the pairwise arithmetic result in the resulting fuzzy component. In other words, the matrix estimates how well each possible combination of discretized input values matches the fuzzy form of the result. The inherited reliability of the result is then obtained as a weighted average of these compatibility values with respect to the latent distributions of the input Z-numbers. A useful feature of the proposed approach is that the α-cuts of the resulting B12 reliability component can be written analytically. For two triangular reliability components, the lower and upper bounds of the result are obtained directly from the corresponding α-level bounds of the original reliability components. This eliminates the need for repeated numerical optimization in the considered problem formulation. Thus, the method has a simple computational structure and can be useful as a base case for more general Z-number arithmetic.

In the present study, the theoretical and numerical discussion is mainly based on unimodal latent probability distributions, since such distributions are more common in practical modeling and usually correspond to a single dominant source or regime of uncertainty. Multimodal distributions, in contrast, often arise when the available information reflects a mixture of several possible regimes, sources, or heterogeneous expert assessments. The proposed lower-prevision representation is compatible with multimodal probability families in principle because it operates on a set of admissible probability measures rather than on a single parametric distribution. Therefore, the same framework can be applied to multimodal latent structures. However, in such cases, the resulting lower and upper bounds may no longer admit simple closed-form expressions and may require numerical optimization over a richer credal set.

Let us also discuss a discretization aspect. Increasing the number of discretization points generally improves the approximation of both the value component (A) and the reliability component (B), as a denser grid captures the shapes of the corresponding membership functions more accurately. However, this improvement comes with a direct increase in computational burden. For two Z-numbers with (n1) and (n2) discretization nodes in their first components, the compatibility structure is represented by a weight matrix with (n1 × n2) elements. Thus, the proposed lower-prevision-based representation reduces complexity compared with directly enumerating all admissible probability distributions, but it does not eliminate the growth caused by discretization itself. Therefore, the choice of discretization level should be understood as a trade-off between numerical accuracy and computational efficiency. The three-point scheme used in this paper is a minimal, transparent case that illustrates the method’s core mechanism, while higher-level discretization may be employed when a more accurate approximation is required.

The triangular approximation of the resulting reliability component B12, considered as an example, is reconstructed from the selected α-levels and then approximately represented as a triangular fuzzy number. This is a practical and convenient representation. The proposed approach is valuable not because it replaces all existing methods of Z-number arithmetic, but because it provides a transparent analytical case. It demonstrates how the reliability component can be propagated through a latent distribution mechanism and how a weight matrix can link fuzzy arithmetic with reliability inheritance. This provides a basis for further extension to more general discretization schemes and more complex operations with Z-numbers. It provides a useful starting point for developing more general and computationally efficient methods for operations with Z-numbers.

The proposed approach should be viewed as a transparent base case rather than a complete solution for all forms of Z-number arithmetic. In its present form, the analytical derivation is limited to triangular fuzzy numbers, a three-point/three-alpha-level discretization scheme, and the addition operation under the independence assumption. Although the lower-prevision framework is, in principle, compatible with more complex settings, such as multimodal latent distributions or denser discretization grids, these cases may require additional numerical optimization and broader computational testing. Extending the method to other arithmetic operations, dependent variables, and non-triangular fuzzy components remains an important direction for future work.

Future research could extend the proposed framework in several directions. First, the method could be generalized to fuzzy numbers with membership functions of different shapes. Second, higher-order discretization schemes could be considered to improve approximation accuracy. Third, other arithmetic operations, such as subtraction, multiplication, and division, could be studied within the same latent distribution framework. Fourth, dependencies between random variables could be incorporated to yield a more flexible reliability propagation model. Another interesting direction for the future work may include relation and synergy of the proposed work with type-2 and type-3 fuzzy sets-based models. Currently, one study [33] examines the relationship between type-2 fuzzy sets and Z-numbers, proving that every monotonic type-2 fuzzy set can be represented as the output of an appropriate data-processing algorithm applied to specific Z-numbers. In view of this, the relationship between type-2 fuzzy sets and Z-numbers, and how our proposed method can be improved by a new version of the type-3 fuzzy system, may be analyzed. In particular, the Type-3 Adaptive Neuro-Fuzzy Inference System With a Noniterative Learning Algorithm [34] could be used as a computation engine to implement the proposed approach once the relationship between type-3 fuzzy sets and Z-numbers has been investigated.

## Figures and Tables

**Table 1:** The comparison results.

Approach	Execution Time, s	Complexity	Main Idea
The proposed approach	0.0000384460	O(1)	The use of envelope of distributions set
Approach in [3]	0.0560225050	O(CLP(n) + m2n2)	All the distributions are treated directly
Approach in [10]	0. 049157	O(Q + r)	Triangular distributions are used

## Data Availability

The original contributions presented in this study are included in the article. Further inquiries can be directed to the corresponding author.
